# Effect of DL-Methylephedrine on Dopamine Transporter Using Positron Emission Tomography With [^18^F]FE-PE2I

**DOI:** 10.3389/fpsyt.2022.799319

**Published:** 2022-05-31

**Authors:** Tsuyoshi Nogami, Ryosuke Arakawa, Takeshi Sakayori, Yumiko Ikeda, Yoshiro Okubo, Amane Tateno

**Affiliations:** ^1^Department of Neuropsychiatry, Nippon Medical School, Tokyo, Japan; ^2^Department of Pharmacology, Nippon Medical School, Tokyo, Japan

**Keywords:** dl-methylephedrine, dopamine transporter, positron emission tomography, [^18^F]FE-PE2I, doping

## Abstract

**Rationale:**

Since ephedrine has a dopamine transporter (DAT) inhibitory effect similar to amphetamine, dl-methylephedrine, a derivative of ephedrine, is considered to have the characteristics of a central nervous system stimulant due to the DAT inhibitory effect. For example, the World Anti-Doping Agency categorizes dl-methylephedrine as a stimulant in the prohibited list for competitions. Assuming to have the same effect as ephedrine, the urinary concentration of dl-methylephedrine is regulated below 10 μg/mL, as is ephedrine. However, the extent to which dl-methylephedrine affects brain function is not yet fully understood.

**Objectives:**

The purpose of this study was to evaluate DAT occupancy by a single oral administration of a daily dose of dl-methylephedrine using positron emission tomography (PET) with [^18^F]FE-PE2I to characterize its stimulatory effect on the central nervous system.

**Methods:**

Nine healthy male volunteers were enrolled in the study. The experiments were designed as a placebo-controlled randomized double-blind crossover comparative study. After the first PET scan in a drug-free state, the second and third PET scans were performed with randomized dosing at 60 mg of dl-methylephedrine or placebo. The plasma and urine concentrations of dl-methylephedrine were measured just before and after the PET scans, respectively.

**Results:**

Mean urine and plasma concentrations of dl-methylephedrine were 13.9 μg/mL and 215.2 ng/mL, respectively. Mean DAT occupancy in the caudate was 4.4% for dl-methylephedrine and 1.2% for placebo. Mean DAT occupancy in the putamen was 3.6% for dl-methylephedrine and 0.5% for placebo. There was no significant difference of DAT occupancies between the groups.

**Conclusion:**

In this study, the urinary concentration of dl-methylephedrine (13.9 μg/mL) was higher than the prohibited reference value (10.0 μg/mL), and there was no significant difference in DAT occupancy between dl-methylephedrine and placebo. These findings suggest that a clinical daily dose of dl-methylephedrine may exceed the doping regulation value according to urine concentration; however, it was considered that at least the central excitatory effect mediated by DAT inhibition was not observed at the daily dose of dl-methylephedrine.

## Introduction

Drug doping in athletics is increasing and diversifying, using drugs that improve not only pure motor function, but also brain functions such as concentration during competition, and such drug usage has become a new problem (1). A wide range of drugs, from prescribed drugs to luxury foods and health foods, which aim to improve brain function, are called nootropic drugs. It is known that modafinil enhances wakefulness and cognitive performance (2, 3), and in recent years nootropic drugs have been used in sports for the purpose of improving competitive ability (4). However, the effects of nootropic drugs on central nervous system function have not been sufficiently investigated in terms of either efficacy or adverse effects.

Ephedrine, dl-methylephedrine, and pseudoephedrine, which are used to treat cough and rhinitis, are classified as stimulants in the World Anti-Doping Agency (WADA) list of prohibited drugs (5). Ephedrine and pseudoephedrine are precursors of methamphetamine, and it is thought that they act as a mechanism of central action in the dopamine system. In addition, since ephedrine has a dopamine transporter (DAT) inhibitory effect similar to amphetamine, dl-methylephedrine, a derivative of ephedrine, is considered to have the characteristics of a central nervous system stimulant due to the DAT inhibitory effect.

DAT controls the spatial and temporal dynamics of dopamine neurotransmission by promoting the reuptake of extracellular transmitter into presynaptic neurons (6). Many of stimulants prohibited by WADA possess DAT inhibition, thus increasing extracellular dopamine. However, it is reported that ephedrine has a weaker, a little <40 times DAT inhibitory action, compared to amphetamine (7), and that pseudoephedrine has a weaker, about 150 times DAT inhibitory action, than amphetamine (8). On the other hand, there is no data regarding any DAT inhibitory action with dl-methylephedrine.

Brain imaging studies are useful to see how psychotropics affect psychiatric disorders (9–11), but there have not been many studies that demonstrate how nootropic drugs affect competitive ability or higher brain function. Some of the nootropic drugs, as well as stimulants, are thought to improve competition ability with effects on neurotransmitters such as choline, dopamine, and serotonin (5). Research with stimulants, such as amphetamine, methylphenidate and ephedrine, showed improved performance (12–14). As these stimulants are thought to improve cognitive function by increasing extracellular dopamine via DAT, it is important to investigate the relation between stimulants and DAT inhibition. DAT inhibition can be evaluated by positron emission tomography (PET) by measuring occupancy. When evaluating drugs in PET study, binding property of drugs administrated to the target molecules such as receptors and transporters is estimated. Binding potentials (BP_ND_) is a quantitative representation of the target combining the density of target molecules to the affinity of a ligand to that target. Occupancy is defined as the treatment-induced change in BP_ND_ following a drug administration. The BP_ND_ value of the PET radioligand decreases when the drug increasingly occupies the target receptor and competes with PET radioligand (15). The advantage of using PET is that it enables intracerebral evaluation with small numbers. For example, in a clinical trial with dose setting of the antipsychotic drug blonanserin, the optimal dose was determined from about 150 participants, but we showed equivalent results with about one-tenth of the participant numbers using PET (16). Several radioligands for imaging DAT have been developed for PET, such as [^11^C]cocaine, [^11^C]β-CIT, and [^11^C]PE2I (17–20). Furthermore, [^18^F]FE-PE2I with high affinity and selectivity for DAT has been developed (21), with kinetics favorable compared to other radioligands, and quantification of DAT is less biased (22, 23). Using this ligand [^18^F]FE-PE2I, several DAT occupancies by stimulants were reported (24, 25).

Some of the stimulants listed by WADA are regulated by urinary concentration levels. For example, cathine is regulated below 5 μg/mL and pseudoephedrine below 150 μg/mL. It is known that pseudoephedrine has a dose-dependent effect on improving athletic ability, and the regulation criteria for pseudoephedrine have been revised based on scientific evidence from research results. Pseudoephedrine was regulated below 25 μg/mL until 2003, was removed from the prohibited drug list between 2004 and 2009, and was then re-entered as a prohibited drug from 2010 (26, 27). Assuming to have the same effect as ephedrine, the urinary concentration of dl-methylephedrine is regulated below 10 μg/mL, the same as ephedrine. However, the extent to which dl-methylephedrine affects brain function is not yet fully understood.

We conducted a DAT occupancy study with dl-methylephedrine using PET with [^18^F]FE-PE2I to characterize its stimulatory effects on the central nervous system.

## Materials and Methods

### Subjects

Ten healthy male volunteers (age range 21–36 years; mean age ± S.D = 27.2 ± 5.2 years; 60 mg) participated in the study. None of the volunteers were excluded due to drug usage. None had a history of present or past psychiatric, neurological or somatic disorders, or alcohol-related problems. All subjects were non-smokers and stopped caffeine intake 48 h prior to the PET scans. The study was approved by the review board of Nippon Medical School Hospital, Japan. After thorough explanation of the study, written informed consent was obtained from all participants.

### Study Design

**Figure 1:**
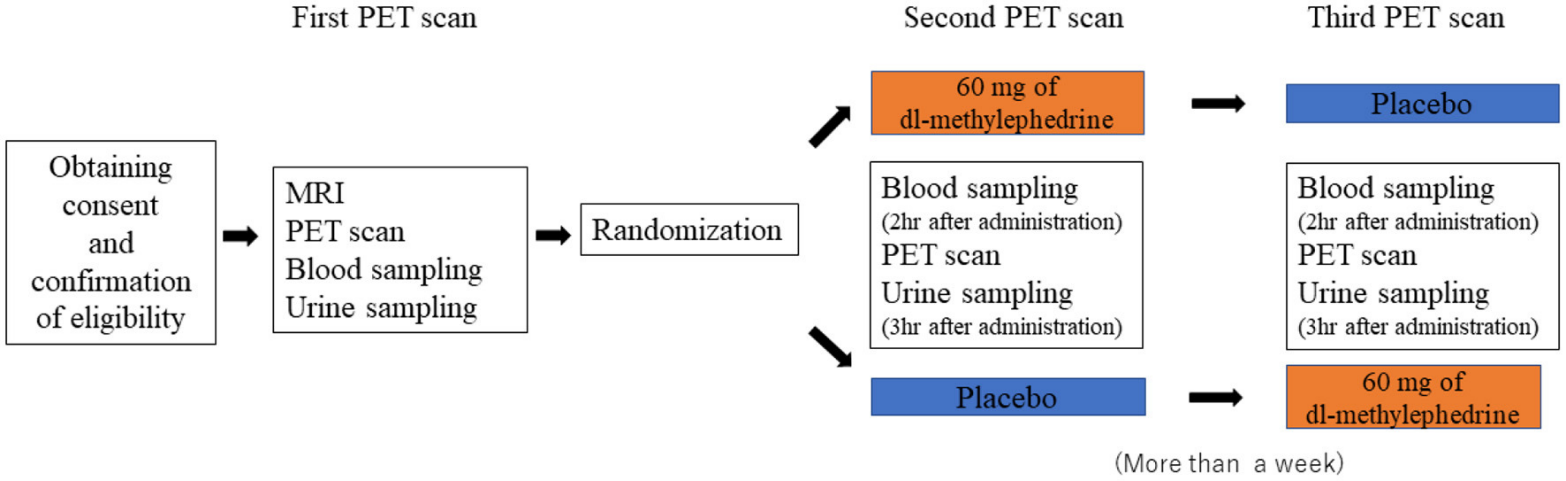
The experimental design of the study.

The experiment was designed as a placebo-controlled randomized double-blind crossover comparative study. Three PET scans were conducted for each subject, with the scans all separated by intervals of more than 1 week. After the first PET scan with drug-free condition, the second and third PET scans were performed with randomized dosing with 60 mg of dl-methylephedrine or placebo (60 mg of lactose). We planned the second and third scans to aim for the T_max_ of dl-methylephedrine, 2 h, which is the time interval after the drug administration to reach the maximum plasma concentration. The plasma and urine concentrations of dl-methylephedrine were measured just before and after the PET scans, respectively (see Figure 1).

### PET Procedures

An Eminence SET-3000GCT-X (Shimadzu Corp., Japan) scanner system was used for all measurements, with a head fixation device to minimize head movement. A 10-min transmission scan was performed to correct for attenuation. Dynamic PET scan was performed for 60 min after intravenous bolus injection of [^18^F]FE-PE2I. Injected radioactivity was 182.9-191.1 (mean ± S.D = 188.2 ± 2.3) MBq at baseline condition, 180.5–189.7 (186.0 ± 2.6) MBq for placebo, and 182.3–190.8 (188.1 ± 2.4) MBq for dl-methylephedrine. Specific radioactivity was 566.8–728.0 (641.7 ± 77.4) GBq/μmol at baseline condition, 581.5–1072.7 (960.4±171.6) GBq/μmol for placebo and 544.0–1072.73 (894.2 ± 313.6) GBq/μmol for dl-methylephedrine.

### MRI Procedure

Magnetic resonance (MR) images of the brain were acquired with 1.5 T MR imaging, Intera 1.5 T Achieva Nova (Philips Medical Systems, Best, Netherlands). T1-weighted MR images were obtained at 1-mm slices. The MRI results revealed no apparent structural abnormalities.

### Plasma and Urine Concentration

Venous blood samples were collected in tubes containing EDTA-2Na, and centrifuged at 3,000 rpm for 10 min at 4°C. Separated plasma samples were stored at −80°C until analysis. Plasma concentration of dl-methylephedrine was measured by a validated method using high-performance liquid chromatography-tandem mass spectrometry (LC-MS/MS) with a target lower quantification limit of 1 ng/mL (LSI Medience Corp., Japan). Urine samples were collected after the PET scans and were stored at −80°C until analysis. The urine concentration of dl-methylephedrine was measured by gas chromatography (LSI Medience Corp., Japan) with a target of lower quantification limit of 0.03 μg/mL.

### Data Analysis

All MR images were co-registered to the PET images using the software package PMOD (version 3.17; PMOD Technologies Ltd, Switzerland). Regions of interest (ROIs) were drawn manually on summed PET images with reference to co-registered MR images and defined for the striatum (caudate and putamen) and cerebellum. The average values of right and left ROIs were used for analysis. DAT bindings were quantified using a simplified reference tissue model (28, 29). The cerebellum was used as reference region because of its negligible DAT density (23). This model allows the estimation of binding potentials (BP_ND_), which were defined as f_ND_ × B_max_/K_d_, where f_ND_ is the free fraction of ligand in the nondisplaceable tissue compartment, B_max_ is the transporter or receptor density, and K_d_ is the dissociation constant (30).

DAT occupancies by dl-methylephedrine and placebo were calculated by the following equation: Occupancy (%) = (BP_baseline_ – BP_drug_)/BP_baseline_ × 100, where Occupancy is DAT occupancy, BP_baseline_ is BP_ND_ in the drug-free state, and BP_drug_ is BP_ND_ after administration of dl-methylephedrine or placebo. Difference of DAT occupancies between dl-methylephedrine and placebo in caudate and putamen were tested by Wilcoxon signed-rank test. The relationship between DAT occupancy of striatum (average of caudate and putamen) and urine concentration with dl-methylephedrine was also estimated by Spearman's rank correlation coefficient.

## Results

**Figure 2:**
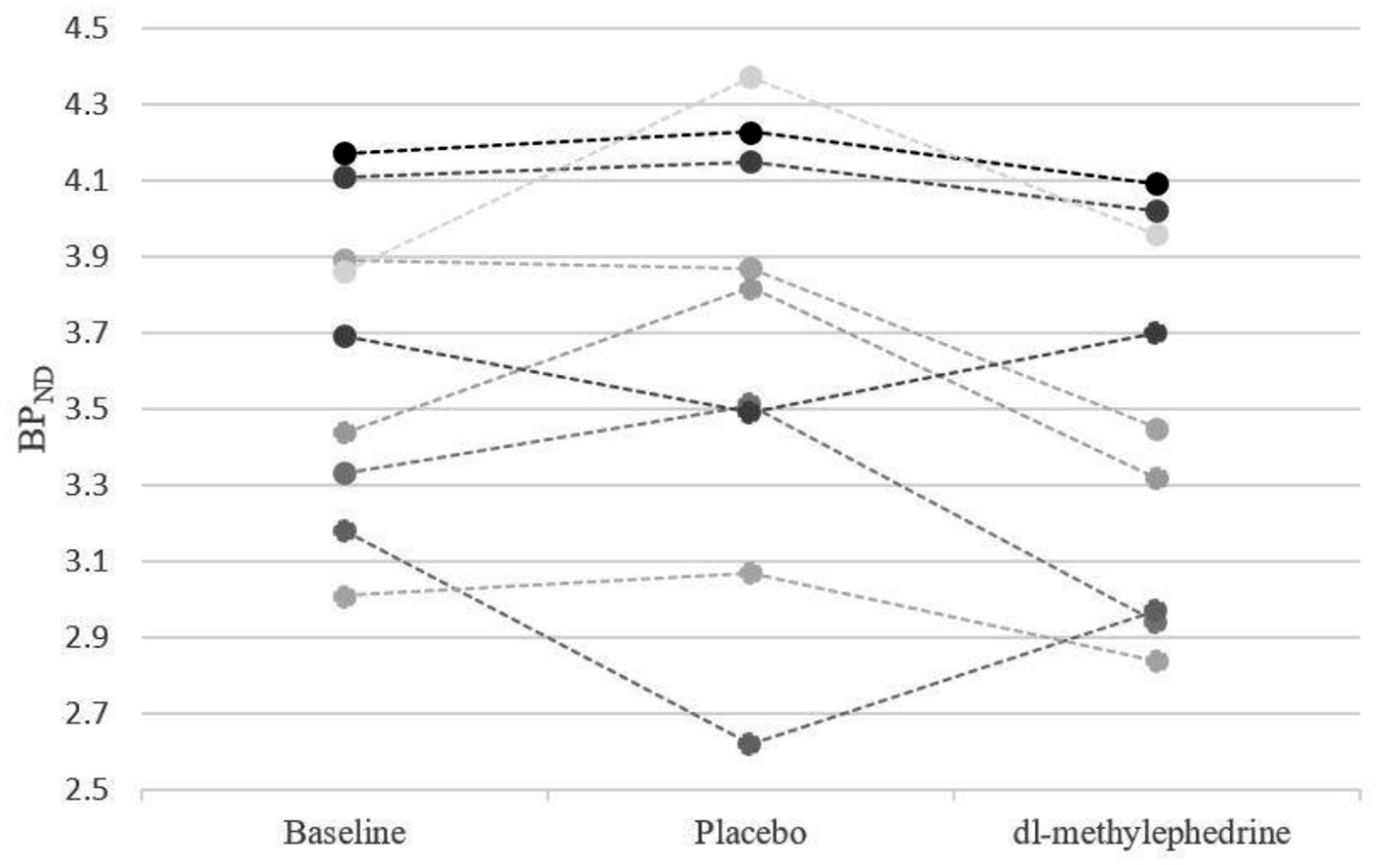
BP_ND_ in caudate for each subject at baseline, placebo, and 60 mg of dl-methylephedrine.

**Figure 3:**
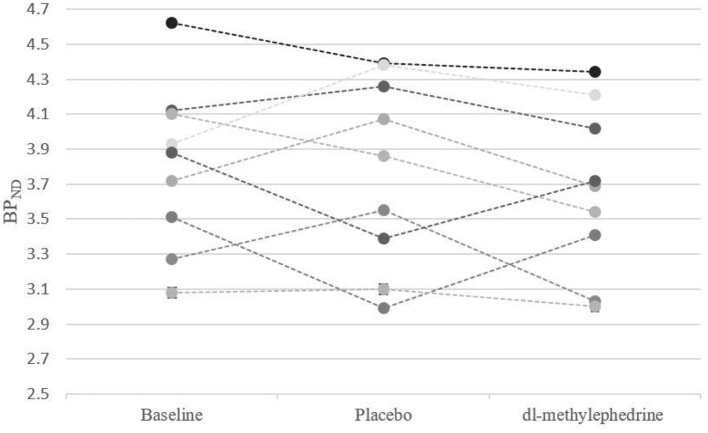
BP_ND_ in putamen for each subject at baseline, placebo, and 60 mg of dl-methylephedrine.

**Figure 4:**
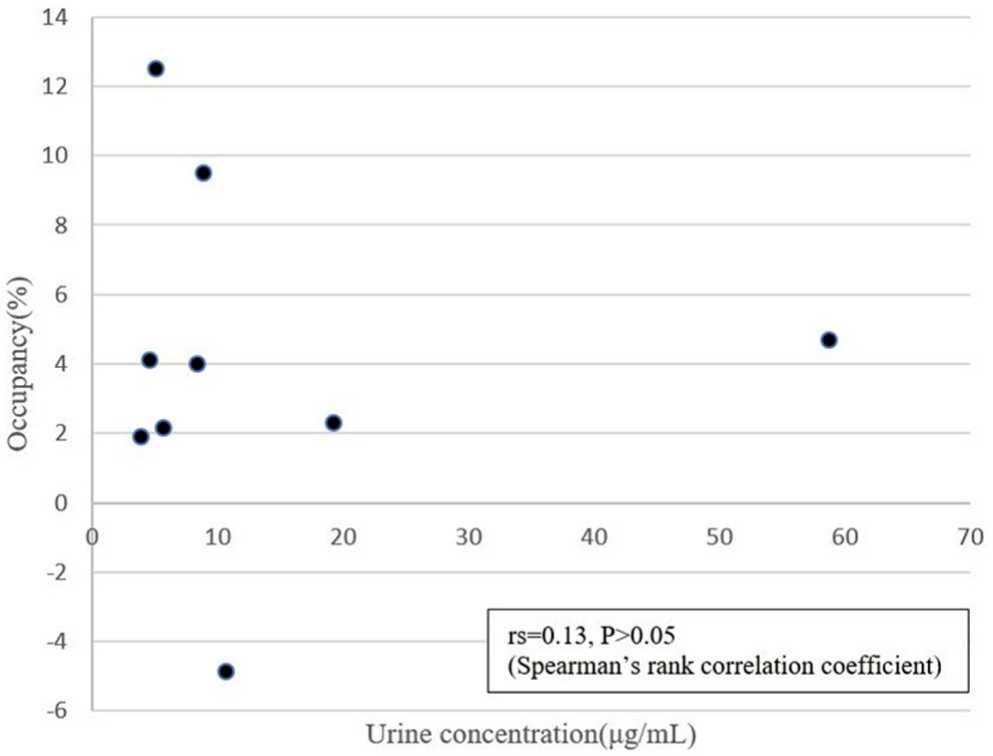
Correlation between urine concentration and DAT occupancy in striatum (average of caudate and putamen) with 60 mg of dl-methylephedrine.

We excluded one volunteer whose BP_ND_ of DAT in the drug-free state was extremely low, as we could not rule out a pre-disease state of neurodegenerative disorders. The remaining 9 were analyzed. BP_ND_ in caudate and putamen are shown in Figures 2, 3. The mean plasma concentration with a single administration of 60 mg dl-methylephedrine was 215.2 ± 97.5 ng/mL (two hours post administration, mean ± S.D; range 73.7–404.9), and mean urine concentration was 13.9 ± 17.5 μg/mL (three hours post administration, mean ± S.D; range 3.86–58.75). Mean ± S.D DAT occupancies in the caudate and putamen measured with [^18^F]FE-PE2I were 4.4 ± 4.9 % and 3.6 ± 5.6 % with 60 mg of dl-methylephedrine and −1.2 ± 9.1 % and 0.5 ± 9.6 % with placebo (Table 1). There was no significant difference between the groups with dl-methylephedrine and placebo [caudate: z = 1.48, *p* = 0.14, putamen: z = 1.01, *p* = 0.31 (Wilcoxon signed-rank test)]. There was no correlation between DAT occupancy in striatum and urine concentration with dl-methylephedrine (rs = 0.13, *P* > 0.05: Spearman's rank correlation coefficient) (Figure 4).

**Table 1:** DAT occupancy in the striatum with placebo and dl-methylephedrine, and blood and urine concentrations of dl-methylephedrine.

**No**.	**Age (yr)**	**BW (Kg)**	**BMI (Kg/m^**2**^)**	**Occupancy (%)**				**Blood concentration (ng/mL)**	**Urine concentration (μg/mL)**
				**Placebo**		**dl-methylephedrine 60 mg**			
				**Caudate**	**Putamen**	**Caudate**	**Putamen**		
1	36	62	21.5	−1	−3.4	2.2	2.4	167.4	19.25
2	22	63	22.9	0.5	5.9	11.3	13.7	245.7	5.09
3	21	60	19.6	−5.4	−8.6	11.7	7.3	264.4	8.89
4	28	66	21.6	−1.4	5	1.9	6.1	189.2	8.33
5	26	59	20.4	−13.2	−11.5	−2.6	−7.1	237.3	10.66
6	27	72	24.9	−11	−9.4	3.5	0.8	249.5	5.7
7	33	58	21.3	17.6	14.8	6.6	2.8	105.2	58.75
8	32	54	19.4	5.4	12.6	−0.3	4.1	73.7	3.86
9	25	50	18.8	−2	−0.6	5.6	2.6	404.9	4.59
Mean	27.8	60.4	21.2	−1.2	0.5	4.4	3.6	215.26	13.9
S.D	5.0	6.4	1.9	9.1	9.6	4.9	5.6	97.5	17.5

*There was no significant difference between the groups with dl-methylephedrine and placebo [caudate: z = 1.48, p = 0.14, putamen: z = 1.01, p = 0.31 (Wilcoxon signed-rank test)]*.

*Blood sample was taken 2 h after administration, and urine sample was taken 3 h after administration BW, body weight; BMI, body mass index*.

## Discussion

To the best of our knowledge, this is the first PET study to evaluate the effect of dl-methylephedrine on DAT in the striatum *in vivo*. Mean DAT occupancy was approximately 4% (4.4% in caudate and 3.6% in putamen) after a single administration of 60 mg dl-methylephedrine. This is remarkably low compared with the results of other prohibited central nervous system stimulants by WADA, such as modafinil, methylphenidate and mazindol. In our previous study, we reported that the DAT occupancy of modafinil with [^18^F]FE-PE2I was 51.4% and 56.9% in the striatum at 200 mg and 300 mg (24). DAT occupancy of methylphenidate with [^11^C]cocaine was reported as 40–74% in the striatum at clinical doses of 10–60 mg (31). As for mazindol, DAT occupancy in the striatum was approximately 25% with 1.5 mg (25). Compared to those results, DAT occupancy by dl-methylephedrine was quite low, approximately 4% in the present study, and there was no significant difference compared with placebo. The test-retest reproducibility of BP_ND_ value estimated by [^18^F]FE-PE2I for the striatum in the previous research was approximately 5% (32). Although the occupancy of the individuals in this study vary to some extent, the average occupancy with placebo was smaller (approximately 1%) than the previous research. Thus, the variety of the results in this study were considered insignificant.

Striatum was evaluated in this study, since DAT density is not high enough to evaluate with PET in other brain regions. Central nervous system stimulants pharmacologically exert their effect by blocking DAT, thereby causing an increase in extracellular dopamine concentrations (33, 34). Dopamine is related to motivation, learning ability, motor ability, memory and reward system (35, 36), and central nervous system stimulants are thought to improve those abilities by increasing extracellular dopamine. There are also some arguments regarding the relationship between abuse liability and dopamine increase by blocking DAT. Cocaine and related compounds bind to DAT increase the extracellular dopamine levels in limbic area, initiates the sequence of events that ultimately cause the rewarding effect. It is well known that the higher binding affinity to the DAT induce the higher rewarding effect (37, 38). Since dl-methylephedrine is a derivative of ephedrine and methamphetamine is synthesized from ephedrine, dl-methylephedrine is also considered to act on the central nervous system via DAT. For this reason, dl-methylephedrine is classified as prohibited drug by WADA.

For the improvement of cognitive function, the brain region other than striatum is also considered to be important. Methylphenidate, used for treatment of attention deficit hyperactivity disorder (ADHD), is also used for the enhancement of cognitive performance (39). Dopamine in prefrontal cortex (PFC) plays a very important role for cognitive functions (40), and it is hypothesized that the improvement of cognitive function by methylphenidate is due to an increase of dopamine in the PFC. However, in a monkey study, methylphenidate showed improvement in various cognitive tasks, with a marked increase in the striatum but no significant difference in PFC, which indicate that improvement of cognitive function is contributed by dopamine increase in striatum or the accompanying change in the PFC-striatal network (41).

To protect the health of athletes and provide them with the opportunity to pursue human excellence without the use of prohibited substances and methods, WADA has issued a prohibited drug list, in which some of the drugs are regulated according to urine concentration. The urine concentration of dl-methylephedrine is regulated at below 10 μg/mL, the same as for ephedrine, whereas pseudoephedrine, an isomer of ephedrine, has a regulated urinary concentration of 150 μg/mL. Pseudoephedrine is widely used as a nonprescription drug in Europe, and investigation was conducted on the regulation values that cause doping effects, resulting in daily administration of doses that did not show changes in performance. Thus, the upper limit of urinary concentration (150 μg/mL) was set as the cutoff value when taken at regular dose (26, 27). In Japan, dl-methylephedrine is included in nonprescription drugs such as cold, rhinitis, and antitussive medications, and the maximal daily dose is 60 to 110 mg. It is cited as one of the causes of inadvertent doping that athletes take to treat cold symptoms. In the present study, the urine concentration 3 h after a single administration of 60 mg dl-methylephedrine was 13.9 μg/mL. Thus, by taking a daily dose of dl-methylephedrine included in the nonprescription medication, the urine concentration may exceed the regulated value. On the other hand, the DAT inhibitory effect by 60 mg of dl-methylephedrine as evaluated by PET and [^18^F]FE-PE2I did not show a significant difference from the placebo. Also, the degree of inhibition was approximately 4%, much lower than the 25–74% of inhibition by other prohibited drugs (24, 25, 31). It is considered that there is no significant dopamine release at this level of DAT occupancy, because in the mazindol study, even with a single administration of 1.5 mg, DAT occupancy was about 25%, but a decrease of BP_ND_, which reflects dopamine release, was only about 2.8 to 4.6% (25).

The results of this study showed that there was no significant DAT inhibition with a single dose of 60 mg of dl-methylephedrine and a urinary concentration over 10 μg/mL. Therefore, the regulated value should be reviewed, as with the current regulation value, the effect on DAT does not differ from that of placebo, with the probability that there is no dopamine release that enhances cognitive ability.

### Limitations and Future Directions

There were some limitations to this study. First, 60 mg of dl-methylephedrine was administered in this study, which is about half of the maximum daily dose, and therefore a study with a higher dose of dl-methylephedrine would be preferred. Second, the sample size participated in this study was small. From the past study, it is unclear how much occupancy makes meaning or is enough to prove doping. Accordingly, in this study, we only calculated the occupancy and evaluated its value without estimating the required sample size using a power analysis. Third, only male volunteers participated in this study. Urine concentration is expected to show higher value with female because of their smaller size. Therefore, the further study with female is needed to evaluate the gender difference.

As the regulation criteria of urine concentration with pseudoephedrine had been revised based on scientific evidence from research results, a direct comparison study with pseudoephedrine to assess the DAT inhibitory effect is needed. We evaluated DAT occupancy in this study, but the effect on other portions of the central nervous system or the relation with cognitive function has not been elucidated; therefore, it is necessary to investigate other neurotransmitters and effects on cognitive function with dl-methylephedrine is beneficial to determine revision of the regulation.

## Conclusion

Dl-methylephedrine is thought to have the feature of a central nervous system stimulant via DAT, but our data suggest that with daily administration, DAT inhibition was quite low in comparison with other prohibited drugs. It was considered that at least the central excitatory effect mediated by DAT inhibition was not observed with a daily dose of dl-methylephedrine. The result indicates that there is room for reconsideration with the regulation for urine concentration of dl-methylephedrine.

## Data Availability Statement

The original contributions presented in the study are included in the article/Supplementary Material, further inquiries can be directed to the corresponding author/s.

## Ethics Statement

The studies involving human participants were reviewed and approved by Review Board of Nippon Medical School Hospital. The patients/participants provided their written informed consent to participate in this study.

## Author Contributions

TN, RA, TS, YI, and AT designed the study, wrote the protocol, and collected the data. YO commented on the protocol. TN and AT analyzed the data. TN wrote the first draft of the manuscript. All authors commented on the manuscript and have approved the final manuscript.

## Funding

This study was partially supported by Japan Sports Agency Ministry of Education, Culture, Sports, Science and Technology-Japan, Research and Development Project on Anti-doping Science.

## Conflict of Interest

RA has received speaker's honoraria from Sumitomo Dainippon Pharma, Meiji, Pfizer. YO has received grants or speaker's honoraria from Sumitomo Dainippon Pharma, GlaxoSmithKline, Janssen Pharmaceutical, Otsuka Pharmaceutical, Pfizer, Eli Lilly, Astellas, Yoshitomi and Meiji. AT has received speaker's honoraria from Dainippon Sumitomo Pharma, Otsuka Pharmaceutical within past 3 years. The remaining authors declare that the research was conducted in the absence of any commercial or financial relationships that could be construed as a potential conflict of interest.

## Publisher's Note

All claims expressed in this article are solely those of the authors and do not necessarily represent those of their affiliated organizations, or those of the publisher, the editors and the reviewers. Any product that may be evaluated in this article, or claim that may be made by its manufacturer, is not guaranteed or endorsed by the publisher.

## References

[1] Smith ACT Stavros C Westberg K. Cognitive enhancing drugs in sport: current and future concerns. Subst Use Misuse. (2020) 55:2064–75. 10.1080/10826084.2020.1775652 32525422

[2] Lyon J. Chess study revives debate over cognition-enhancing drugs. JAMA. (2017) 318:784–6. 10.1001/jama.2017.8114 28813563

[3] Lynch G Palmer LC Gall CM. The likelihood of cognitive enhancement. Pharmacol Biochem Behav. (2011) 99:116–29. 10.1016/j.pbb.2010.12.024 21215768 PMC3114293

[4] C. Chiamulera. Research is needed on the use of cognitive enhancer drugs in sport. J Sci Med Sport. (2011) 14:2–3. 10.1016/j.jsams.2010.09.001 20951088

[5] Docherty JR. Pharmacology of stimulants prohibited by the World Anti-Doping Agency (WADA). Br J Pharmacol. (2008) 154:606–22. 10.1038/bjp.2008.124 18500382 PMC2439527

[6] Vaughan RA Foster JD. Mechanisms of dopamine transporter regulation in normal and disease states. Trends Pharmacol Sci. (2013) 34:489–96. 10.1016/j.tips.2013.07.005 23968642 PMC3831354

[7] Jitca G Osz BE Tero-Vescan A Vari CE. Psychoactive drugs-from chemical structure to oxidative stress related to dopaminergic neurotransmission. a review. Antioxidants (Basel). (2021) 10:381. 10.3390/antiox10030381 33806320 PMC8000782

[8] Ruksee N Tongjaroenbuangam W Casalotti SO Govitrapong P. Amphetamine and pseudoephedrine cross-tolerance measured by c-Fos protein expression in brains of chronically treated rats. BMC Neurosci. (2008) 9:99. 10.1186/1471-2202-9-99 18834549 PMC2567327

[9] Sakayori T Tateno A Arakawa R Kim WC Okubo Y. Evaluation of dopamine D3 receptor occupancy by blonanserin using [(11)C]-(+)-PHNO in schizophrenia patients. Psychopharmacology (Berl). (2021) 238:1343–50. 10.1007/s00213-020-05698-3 33180175 PMC8062348

[10] Arakawa R Stenkrona P Takano A Svensson J Andersson M Nag S. Venlafaxine ER blocks the norepinephrine transporter in the brain of patients with major depressive disorder: a PET study using [18F]FMeNER-D2. Int J Neuropsychopharmacol. (2019) 22:278–5. 10.1093/ijnp/pyz003 30649319 PMC6441126

[11] Nogami T Takano H Arakawa R Ichimiya T Fujiwara H Kimura Y. Occupancy of serotonin and norepinephrine transporter by milnacipran in patients with major depressive disorder: a positron emission tomography study with [(11)C]DASB and (S,S)-[(18)F]FMeNER-D(2). Int J Neuropsychopharmacol. (2013) 16:937–43. 10.1017/S1461145712001009 23067569

[12] Chandler JV Blair SN. The effect of amphetamines on selected physiological components related to athletic success. Med Sci Sports Exer. (1980) 12:65–9. 10.1249/00005768-198021000-00013 7392905

[13] Swart J Lamberts RP Lambert MI St Clair Gibson A Lambert EV Skowno J. Exercising with reserve: evidence that the central nervous system regulates prolonged exercise performance. Br J Sports Med. (2009) 43:782–8. 10.1136/bjsm.2008.055889 19052141

[14] Bell DG Jacobs I Ellerington K. Effect of caffeine and ephedrine ingestion on anaerobic exercise performance. Med Sci Sports Exerc. (2001) 33:1399–403. 10.1097/00005768-200108000-00024 11474345

[15] Arakawa R Takano A Halldin C. PET technology for drug development in psychiatry. Neuropsychopharmacol Rep. (2020) 40:114–21. 10.1002/npr2.12084 32463584 PMC7722687

[16] Tateno A Arakawa R Okumura M Fukuta H Honjo K Ishihara K. Striatal and extrastriatal dopamine D2 receptor occupancy by a novel antipsychotic, blonanserin: a PET study with [11C]raclopride and [11C]FLB 457 in schizophrenia. J Clin Psychopharmacol. (2013) 33:162–9. 10.1097/JCP.0b013e3182825bce 23422369

[17] Fowler KJ Saffery R Kile BT Irvine DV Hudson DF Trowell HE. Genetic mapping of mouse centromere protein (Incenp and Cenpe) genes. Cytogenet Cell Genet. (1998) 82:67–70. 10.1159/000015066 9763662

[18] Farde L Halldin C Muller L Suhara T Karlsson P Hall H. PET study of [11C]beta-CIT binding to monoamine transporters in the monkey and human brain. Synapse. (1994) 16:93–103. 10.1002/syn.890160203 8197578

[19] Halldin C Erixon-Lindroth N Pauli S Chou YH Okubo Y Karlsson P. [(11)C]PE2I: a highly selective radioligand for PET examination of the dopamine transporter in monkey and human brain. Eur J Nucl Med Mol Imaging. (2003) 30:1220–30. 10.1007/s00259-003-1212-3 12811422

[20] Emond P Garreau L Chalon S Boazi M Caillet M Bricard J. Synthesis and ligand binding of nortropane derivatives: N-substituted 2beta-carbomethoxy-3beta-(4'-iodophenyl)nortropane and N-(3-iodoprop-(2E)-enyl)-2beta-carbomethoxy-3beta-(3',4'-disubstituted phenyl)nortropane. New high-affinity and selective compounds for the dopamine transporter. J Med Chem. (1997) 40:1366–72. 10.1021/jm960795d 9135033

[21] Varrone A Steiger C Schou M Takano A Finnema SJ Guilloteau D. In vitro autoradiography and in vivo evaluation in cynomolgus monkey of [18F]FE-PE2I, a new dopamine transporter PET radioligand. Synapse. (2009) 63:871–80. 10.1002/syn.20670 19562698

[22] Varrone A Toth M Steiger C Takano A Guilloteau D Ichise M. Kinetic analysis and quantification of the dopamine transporter in the nonhuman primate brain with 11C-PE2I and 18F-FE-PE2I. J Nucl Med. (2011) 52:132–9. 10.2967/jnumed.110.077651 21189414

[23] Sasaki T Ito H Kimura Y Arakawa R Takano H Seki C. Quantification of dopamine transporter in human brain using PET with 18F-FE-PE2I. J Nucl Med. (2012) 53:1065–73. 10.2967/jnumed.111.101626 22689927

[24] Kim W Tateno A Arakawa R Sakayori T Ikeda Y Suzuki H. In vivo activity of modafinil on dopamine transporter measured with positron emission tomography and [(1)(8)F]FE-PE2I. Int J Neuropsychopharmacol. (2014) 17:697–703. 10.1017/S1461145713001612 24451483

[25] Kimura Y Maeda J Yamada M Takahata K Yokokawa K Ikoma Y. et al. Measurement of psychological state changes at low dopamine transporter occupancy following a clinical dose of mazindol. Psychopharmacology (Berl). (2017) 234:323–8. 10.1007/s00213-016-4464-x 27766370

[26] Pokrywka A Tszyrsznic W Kwiatkowska DJ. Problems of the use of pseudoephedrine by athletes. Int J Sports Med. (2009) 30:569–72. 10.1055/s-0029-1202826 19382058

[27] Trinh KV Kim J Ritsma A. Effect of pseudoephedrine in sport: a systematic review. BMJ Open Sport Exerc Med. (2015) 1:e000066. 10.1136/bmjsem-2015-000066 27900142 PMC5117033

[28] Ito H Sudo Y Suhara T Okubo Y Halldin C Farde L. Error analysis for quantification of [(11)C]FLB 457 binding to extrastriatal D(2) dopamine receptors in the human brain. Neuroimage. (2001) 13:531–9. 10.1006/nimg.2000.0717 11170818

[29] Lammertsma AA Hume SP. Simplified reference tissue model for PET receptor studies. Neuroimage. (1996) 4:153–8. 10.1006/nimg.1996.0066 9345505

[30] Innis RB Cunningham VJ Delforge J Fujita M Gjedde A Gunn RN. Consensus nomenclature for in vivo imaging of reversibly binding radioligands. J Cereb Blood Flow Metab. (2007) 27:1533–9. 10.1038/sj.jcbfm.9600493 17519979

[31] Volkow ND Wang GJ Fowler JS Gatley SJ Logan J Ding YS. Dopamine transporter occupancies in the human brain induced by therapeutic doses of oral methylphenidate. Am J Psychiatry. (1998) 155:1325–31. 10.1176/ajp.155.10.1325 9766762

[32] Suzuki M Ito H Kodaka F Takano H Kimura Y Fujiwara H. Reproducibility of PET measurement for presynaptic dopaminergic functions using L-[beta-(11)C]DOPA and [(18)F]FE-PE2I in humans. Nucl Med Commun. (2014) 35:231–7. 10.1097/MNM.0000000000000052 24468851

[33] Howell LL Kimmel HL. Monoamine transporters and psychostimulant addiction. Biochem Pharmacol. (2008) 75:196–217. 10.1016/j.bcp.2007.08.003 17825265

[34] Mortensen OV Amara SG. Dynamic regulation of the dopamine transporter. Eur J Pharmacol. (2003) 479:159–70. 10.1016/j.ejphar.2003.08.066 14612147

[35] Berke JD. What does dopamine mean? Nat Neurosci. (2018) 21:787–93. 10.1038/s41593-018-0152-y 29760524 PMC6358212

[36] Salamone JD. Complex motor and sensorimotor functions of striatal and accumbens dopamine: involvement in instrumental behavior processes. Psychopharmacology (Berl). (1992) 107:160–74. 10.1007/BF02245133 1615120

[37] Greenhill LL. The science of stimulant abuse. Pediatr Ann. (2006) 35:552–6. 10.3928/0090-4481-20060801-07 16986449

[38] Volkow ND Fowler JS Wang GJ Baler R Telang F. Imaging dopamine's role in drug abuse and addiction. Neuropharmacology. (2009) 56 (Suppl 1):3–8. 10.1016/j.neuropharm.2008.05.022 18617195 PMC2696819

[39] Mehta MA Owen AM Sahakian BJ Mavaddat N Pickard JD Robbins TW. Methylphenidate enhances working memory by modulating discrete frontal and parietal lobe regions in the human brain. J Neurosci. (2000) 20:RC65. 10.1523/JNEUROSCI.20-06-j0004.2000 10704519 PMC6772505

[40] Robbins TW Arnsten AF. The neuropsychopharmacology of fronto-executive function: monoaminergic modulation. Annu Rev Neurosci. (2009) 32:267–87. 10.1146/annurev.neuro.051508.135535 19555290 PMC2863127

[41] Kodama T Kojima T Honda Y Hosokawa T Tsutsui KI Watanabe M. Oral administration of methylphenidate (Ritalin) affects dopamine release differentially between the prefrontal cortex and striatum: a microdialysis study in the monkey. J Neurosci. (2017) 37:2387–94. 10.1523/JNEUROSCI.2155-16.2017 28154152 PMC6596846

