# Supplementary figures and images for: Effect of DL-Methylephedrine on Dopamine Transporter Using Positron Emission Tomography With [18F]FE-PE2I
Source: Front Psychiatry. 2022 May 31;13:799319. doi: 10.3389/fpsyt.2022.799319 (PMC9193582; doi:10.3389/fpsyt.2022.799319)

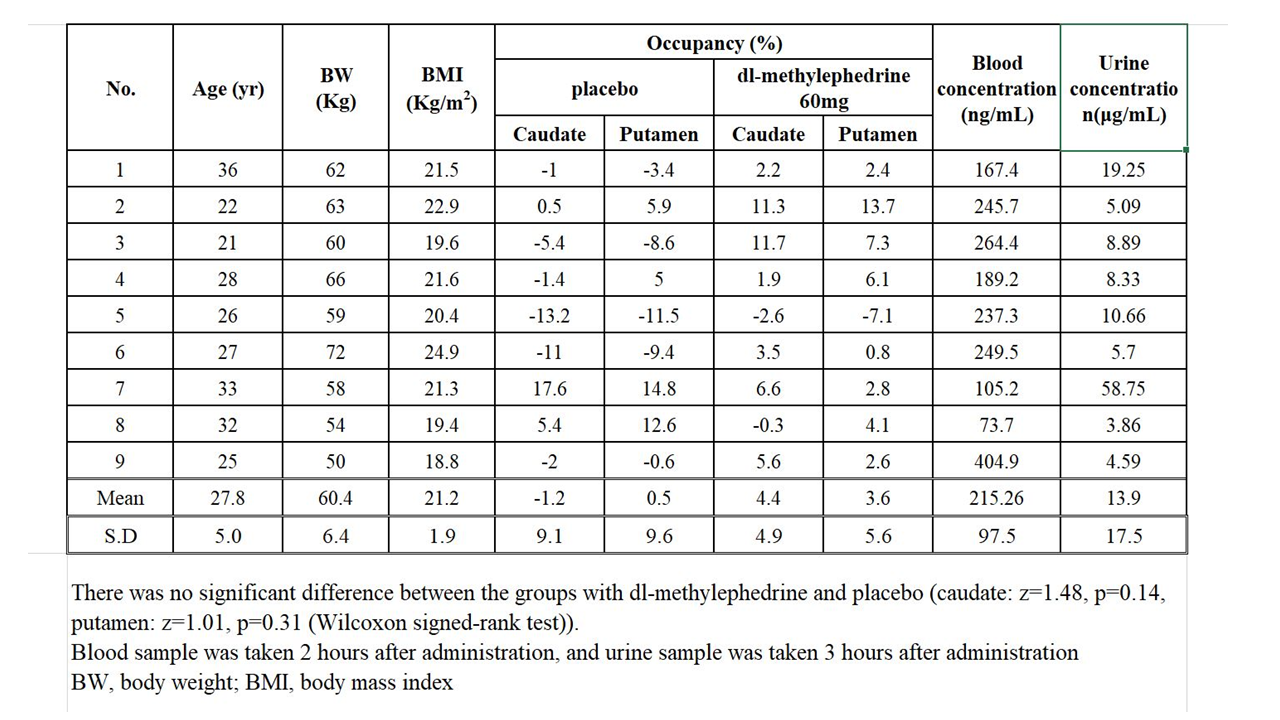

Supplement: Supplementary file 1 [file Image_1.TIF]
